# Opportunities for engineering outer membrane vesicles using synthetic biology approaches

**DOI:** 10.20517/evcna.2023.21

**Published:** 2023-06-08

**Authors:** Richard J. R. Kelwick, Alexander J. Webb, Paul S. Freemont

**Affiliations:** ^1^Section of Structural and Synthetic Biology, Department of Infectious Disease, Imperial College London, London SW7 2AZ, UK.; ^2^UK Dementia Research Institute Care Research and Technology Centre, Imperial College London, Hammersmith Campus, London W12 0NN, UK.; ^3^The London Biofoundry, Imperial College Translation & Innovation Hub, White City Campus, London W12 0BZ, UK.; ^#^Authors contributed equally.

**Keywords:** Outer membrane vesicles, OMVs, synthetic biology, extracellular vesicles, therapeutics, diagnostics, vaccines, microbiology

## Abstract

Gram-negative bacteria naturally shed lipid vesicles, which contain complex molecular cargoes, from their outer membrane. These outer membrane vesicles (OMVs) have important biological functions relating to microbial stress responses, microbiome regulation, and host-pathogen interactions. OMVs are also attractive vehicles for delivering drugs, vaccines, and other therapeutic agents because of their ability to interact with host cells and their natural immunogenic properties. OMVs are also set to have a positive impact on other biotechnological and medical applications including diagnostics, bioremediation, and metabolic engineering. We envision that the field of synthetic biology offers a compelling opportunity to further expand and accelerate the foundational research and downstream applications of OMVs in a range of applications including the provision of OMV-based healthcare technologies. In our opinion, we discuss how current and potential future synergies between OMV research and synthetic biology approaches might help to further accelerate OMV research and real-world applications for the benefit of animal and human health.

Gram-negative bacteria naturally shed lipid vesicles, which contain complex molecular cargoes, from their outer membrane^[1]^. These outer membrane vesicles (OMVs) have diverse and important biological functions relating to microbial stress responses, and play a crucial role in intra- and inter-bacterial communication for microbiome regulation and host-pathogen interactions including immunomodulatory functions^[2,3]^. Essentially, OMVs can enable Gram-negative bacteria to respond to, and somewhat influence, their microenvironment^[4]^. Gram-positive bacteria (e.g., *Bacillus subtilis*) and mycolic acid-containing bacteria (e.g., *Mycobacterium* and *Corynebacterium*) also produce different types of membrane vesicles (MVs)^[5,6]^, although these are not the focus of our opinion. Beyond their natural biological functions, OMVs might also serve biotechnological applications and are therefore being developed as therapeutics, human or animal vaccines, medical imaging and biosensing agents, or as scaffolds for metabolic engineering or bioremediation^[7-14]^. Mechanisms relating to OMV biogenesis/formation, and their molecular compositions (lipid, protein, nucleic acids, and small molecules) are being studied across many different bacteria and culture contexts (e.g., natural environment or bioreactor fermentation)^[1]^. This foundational understanding will likely be beneficial to the long-term development of OMV-based biotechnological applications. It is our opinion that synthetic biology bioengineering approaches could also help accelerate OMV foundational research and OMV-based biotechnological applications including the provision of OMV-based healthcare technologies^[8,10,15-17]^.

Synthetic biology has emerged during the last several decades as an exciting interdisciplinary scientific field that seeks to systematically address biological complexity and to rationally engineer biological systems for useful purposes^[18,19]^. To this end, the field has established a suite of cutting-edge methodologies and tools, underpinned by an engineering framework and responsible innovation practices, that have helped accelerate many real-world applications^[20-23]^. On a fundamental level, synthetic biology employs an engineering framework around the concept of the design-build-test-learn (DBTL) cycle or the synthetic biology design cycle^[18,22,24-27]^ [Figure 1]. The design cycle allows the optimisation of rationally designed biotechnologies and provides a strategy to address biological complexity^[18,19,27]^. Implicit within this framework is a focus on standardised experimental protocols and rigorous biological metrology^[28,29]^. This rigorous approach is also shared by the wider international extracellular vesicle research community in the form of research standards guidelines (e.g., MISEV2018) or technical research papers from the community^[30,31]^. However, we feel that further multi-disciplinary learning between the synthetic biology and EV fields regarding experimental design, protocols, research tools, and biological metrology would be beneficial to both fields. For example, synthetic biology has greatly expanded the throughput of the design cycle using automation (e.g., acoustic and liquid handling robotics platforms) to set up large-scale, multiparameter experiments^[19,22,27,32]^. These approaches reduce errors associated with manual pipetting and produce larger datasets that, especially in combination with design-of-experiment (DOE) or artificial intelligence (AI)-guided methodologies, can lead to deeper biological insights more quickly than conventional biological research workflows^[33,34]^.

**Figure 1:**
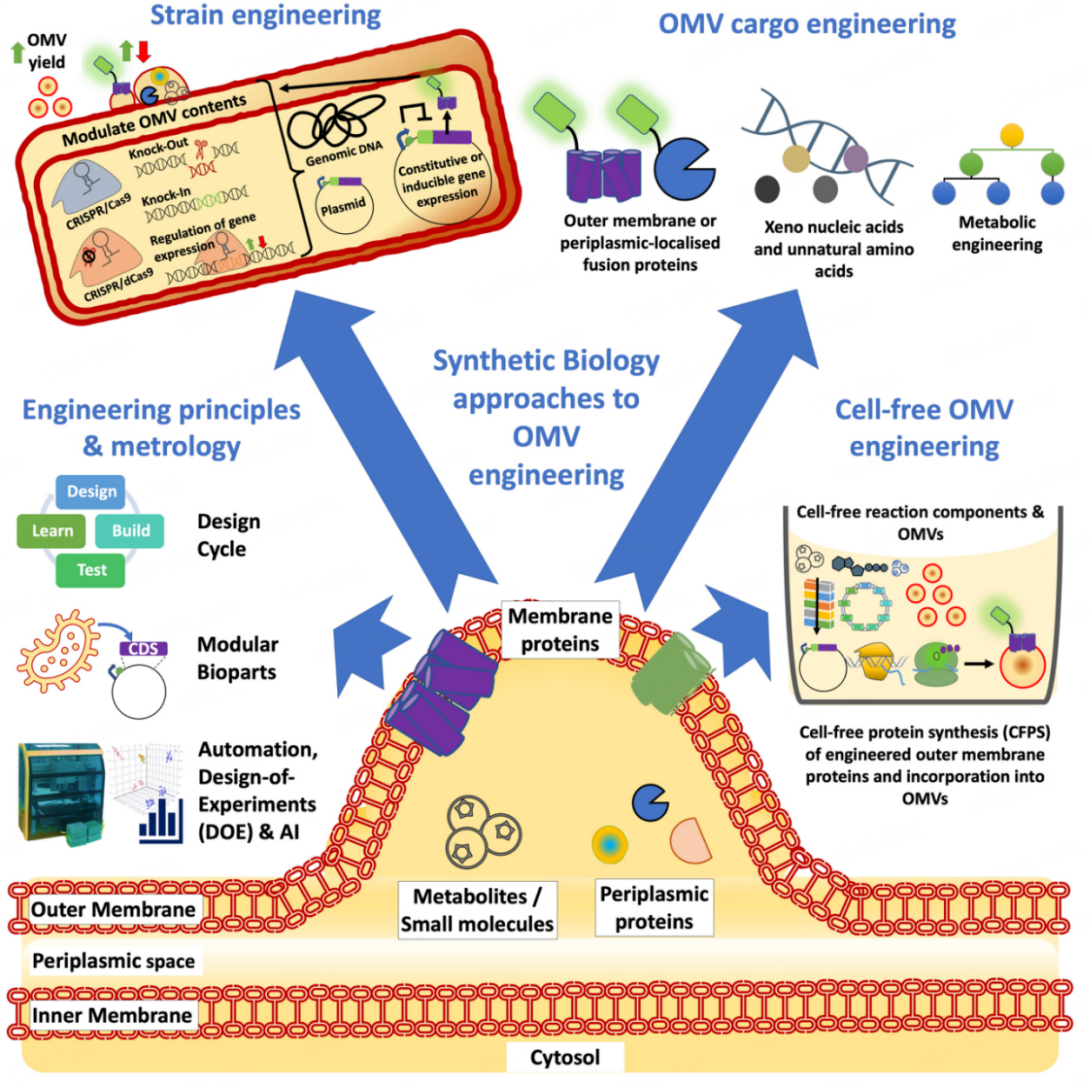
Synthetic biology approaches to outer membrane vesicle (OMV) engineering. The figure depicts synthetic biology engineering approaches and example biotechnologies that could be utilised to engineer OMV producing strains to improve OMV yields and/or the therapeutic cargos of microbially produced OMVs. AI: artificial intelligence; CDS: coding sequence; CRISPR/dCas9: clustered regularly interspaced short palindromic repeats (CRISPR)/endonuclease deficient CRISPR-associated protein 9 (dCas9); DOE: design of experiments; OMV: outer membrane vesicle.

In an OMV engineering context, a DBTL-cycle approach could be employed to systematically engineer bacterial strains with altered OMV cargoes. An interesting and relevant example of this was demonstrated by Zanella *et al.* in which they used a CRISPR/Cas9-based genome editing approach to systematically knock out 59 endogenous OMV-cargo protein genes in an engineered BL21(DE3)Δ60 *Escherichia coli* strain^[15]^. This study not only provided foundational insights into endogenous OMV protein cargo loading in *E. coli* BL21, but also demonstrated an engineering strategy to increase the level of recombinant proteins that can be loaded into the strains OMVs. These insights could be exploited to produce more effective OMV-based vaccines. Complementary to this approach, Alves *et al.* demonstrated that phosphotriesterase (PTE)-SpyCatcher and SpyTagged-OmpA transmembrane fusion proteins facilitated efficient packaging of PTE enzymes within OMVs^[11]^, thereby expanding the utility of this important synthetic biology tool as a bioconjugation system for OMV engineering applications. While in another study, Eastwood *et al.* engineered a vesicle nucleating peptide derived from human α-synuclein to efficiently load a panel of OMV cargo proteins^[35]^. Such approaches could also conceivably facilitate more efficient loading of Cas9 into OMVs for medical applications. For example, OMVs have been utilised as a mechanism for delivering Cas9 to human microbial pathogens to elicit targeted and potent DNA damage. This route has been posited as a potential future therapeutic strategy to combat antimicrobial resistance^[36]^. It is also apparent that synthetic biology is developing many other genome editing tools [e.g., Transcription activator-like effector nucleases (TALENs), zinc-finger nucleases (ZFNs) and nucleobase deaminase enzymes]^[37]^, and gene expression regulation technologies (e.g., catalytically dead CRISPR/dCas9) [Figure 1]^[38,39]^ that could also be applied in future OMV engineering studies. One important application might be the use of sophisticated strain engineering approaches to finely tune bacterial/OMV lipopolysaccharide (LPS) content, the surface display of engineered polysaccharide antigens or the content of other immunomodulatory molecules to minimise unwanted cytotoxicity and maximise OMV vaccine efficacy^[13-15,17,40,41]^. Alternatively, OMVs from multiple different strains could also be pooled together to improve vaccine efficacy. Indeed, an OMV pooling strategy was recently employed to develop a poultry vaccine against avian pathogenic *E. coli* (APEC)^[14]^. OMV vaccines for human health have also been developed, including Bexsero^®^, a *Neisseria meningitidis* vaccine, that has received US FDA approval^[10]^. Other human and animal OMV-based vaccines are also in development^[10,14]^.

Synthetic biology has also greatly expanded the number of gene regulatory elements (e.g., promoters) and other functional genetic elements (e.g., periplasmic localisation tags)^[26,42]^, which, along with their modular (re-useable) nature, and potential for compatibility with high-throughput DNA assembly methods (e.g., Golden Gate)^[18,43]^, creates almost endless possibilities for engineering OMV-producing strains with bespoke molecular cargoes. Furthermore, cell-free protein synthesis systems (CFPS), which utilise isolated cellular transcription/translation machinery, could be used to prototype and test many different assembled expression plasmids or cargo designs to accelerate future OMV engineering design cycles^[22,44]^ [Figure 1]. Furthermore, recent innovations in protein design and folding, including AlphaFold^[45]^, protein large language models (e.g., ESM-2)^[46]^, and other powerful protein structure/function design tools^[47,48]^, could be applied to future OMV studies to engineer entirely *de novo* designed OMV cargo or membrane fusion-proteins. By extension, recent advancements in bacterial metabolic engineering strategies^[12,49]^, including codon reassignment and non-natural amino acid incorporation^[50-52]^, and xeno nucleic acids (XNAs)^[53]^, may lead to powerful OMV cargoes and therapeutic modalities that are entirely synthetic and orthogonal to the production host-cells’ biochemistry. In the near future, the convergence of synthetic biology technologies with OMV engineering approaches may lead to the emergence of synthetic membrane vesicles (MVs) from entirely engineered cells^[54,55]^.

This leads to the interesting question of whether synthetic cell-derived MVs might also serve as intercellular communication vehicles to coordinate synthetic cell consortia. While significant technical challenges remain before synthetic cell-derived MVs become routine, there is scope for fruitful collaborations between OMV researchers and the synthetic cell communities. For example, improvements in methods to exogenously load small molecule, protein or nucleic acid cargoes into lipid vesicles, whether they are OMVs or synthetic cells, will be useful to both fields^[19,55-57]^. Indeed, it should also be noted that the origins of future exogenous cargo molecules might also be the product of synthetic biology-based manufacturing processes^[12,19,23]^. Contemporary OMV engineering efforts are already making an impact across disparate applications. For example, OMVs loaded with Gentamicin, the receptor binding domain of the SARS-CoV-2 spike protein, 5-Fluorouracil (5-FU), chlorin e6 (Ce6), Doxorubicin (DOX), Keratinocyte Growth Factor-2 (KGF-2), melanin, therapeutic siRNAs or other molecules hold promise as future infectious disease or cancer therapeutics, respectively^[7,9,13,16,17,58]^. Engineered OMVs with tumour targeting and imaging/biosensing modalities have also been described^[7]^ with clear implications for future OMV-based medical diagnostics. OMV metabolic engineering strategies are also being developed including a notable example by Yang, Park and Lee in which they metabolically engineered *E. coli* strains to produce colourants that could be used in the food, cosmetic, chemical, or pharmaceutical industries^[12]^. Their use of OMV engineering approaches was integral to the optimisation of the rainbow colourant production process. OMVs can also serve as enzyme display scaffolds to improve the efficiency of enzymatic cascade reactions in biomanufacturing or bioremediation processes^[7]^. Importantly, these studies serve as examples of the powerful synergies that are possible between synthetic biology and OMV research in the context of industrial or therapeutic biomanufacturing.

## CONCLUSION

OMVs hold great promise as future therapeutics, vaccines, diagnostics, and industrial or pharmaceutical manufacturing agents. Indeed, several OMV-based vaccines are already in use. We envision that future convergences between synthetic biology and OMV research will likely expand future OMV-based applications. However, there are foundational knowledge gaps in our understanding of OMV molecular heterogeneity and biogenesis in different contexts. Furthermore, manufacturing OMVs at suitable yields, purity and bioactivities is also challenging and may require additional innovations in OMV isolation technologies, engineering approaches and OMV characterisation methods. However, we envision that a combination of synthetic biology and OMV tools and research approaches will help both fields to overcome these challenges, thereby accelerating the translation of OMVs toward additional real-world applications for the benefit of animal and human health.

## DECLARATIONS

### Copyright

© The Author(s) 2023.
